# Dengue Hemorrhagic Fever Complicated With Hemophagocytic Lymphohistiocytosis in an Adult With Diabetic Ketoacidosis

**DOI:** 10.7759/cureus.10172

**Published:** 2020-08-31

**Authors:** Ethayakumar Narayanasami, Maheswaran Umakanth, Navaneethakrishnan Suganthan

**Affiliations:** 1 Medicine, Base Hospital, Batticaloa, LKA; 2 Clinical Medicine, Eastern University Sri Lanka, Batticaloa, LKA; 3 University Medical Unit, Teaching Hospital, Batticaloa, LKA; 4 Medicine, University of Jaffna, Jaffna, LKA; 5 University Medical Unit, Teaching Hospital, Jaffna, LKA

**Keywords:** dengue, dengue hemorrhagic fever, hemophagocytic lymphohistiocytosis (hlh), diabetic ketoacidosis

## Abstract

Dengue infection can cause a wide spectrum of presentations extending from simple self-limiting febrile illness to severe dengue, including dengue hemorrhagic fever (DHF) and dengue shock syndrome (DSS). Dengue associated hemophagocytic lymphohistiocytosis (HLH) is a rare, life-threatening condition characterized by the uncontrolled activation of macrophages and T cells, eliciting clusters of symptoms and signs and abnormal biochemical parameters. Herein we report a 28-year-old Sri Lankan female with no past medical history who presented with dengue hemorrhagic fever and diabetic ketoacidosis complicated with hemophagocytic lymphohistiocytosis. She was treated with a three-day course of intravenous methylprednisolone in addition to standard care for diabetic ketoacidosis and dengue hemorrhagic fever. She made an uneventful recovery.

## Introduction

Dengue fever (DF) is the most rapidly spreading mosquito-borne viral illness transmitted by *Aedes *mosquitos, particularly *Aedes aegypti* and *Aedes albopictus*. Dengue virus infection is widely distributed in the tropical and subtropical regions of the globe, affecting up to 100 million people annually; 2.5 billion people are at risk [1]. It is endemic in Southeast Asia, including Sri Lanka. Furthermore, with the growing incidence of DF, an increasing number of cases are being reported with rare complications and atypical presentations [2,3].

Dengue fever (DF) can cause a wide spectrum of presentations extending from simple self-limiting febrile illness to severe dengue, including dengue hemorrhagic fever (DHF) and dengue shock syndrome (DSS). However, unusual complications such as acute hepatic failure, acute renal failure, dengue encephalitis, myocarditis, and hematological complications such as hemophagocytic lymphohistiocytosis (HLH), disseminated intravascular coagulopathy have been reported worldwide [4,5].

Moreover, other unusual hematological complications following dengue fever, such as idiopathic thrombocytopenic purpura (ITP) and aplastic anemia, have also been described.

HLH is a rare but potentially lethal hematologic disorder characterized by hyper inflammation, uncontrolled proliferation of activated lymphocytes, prolonged fever, pancytopenia, jaundice, and hepatosplenomegaly [5]. Herein we describe a case of dengue hemorrhagic fever complicated with hemophagocytic lymphohistiocytosis in an adult with diabetic ketoacidosis.

## Case presentation

A 28-year-old, previously healthy, Sri Lankan female presented to the emergency department with a history of high-grade fever of six days, which was associated with myalgia, arthralgia, and retroorbital pain. She also admitted that she had diffuse abdominal pain, loss of appetite, and vomiting for the last two days. However, she denied a history of diarrhea, distention of the abdomen, or mucocutaneous bleeding. She had no history suggestive of either upper or lower respiratory tract infection or meningitis. Although she had no symptoms of urinary tract infection, she noticed that she had been voiding unusually large volume of urine despite inadequate oral intake for a couple of days prior to admission.

On arrival to the accident and emergency department, she was alert, her Glasgow coma scale (GCS) was 15/15, and she was severely dehydrated. The rest of the general examination showed no abnormality except tachypnoea with a respiratory rate of 28 cycles per minute, SpO2 of 95% on ambient air, and a temperature of 39 degrees Celsius. Cardiovascular examination revealed a pulse rate of 128 beats per minute and blood pressure of 100/90mmHg with a significant postural drop in the sitting position. There was no murmur or added heart sound. There was mild, tender hepatomegaly on the abdominal examination with no free fluid. Respiratory system examination showed reduced breath sound with stony dullness over the right base compatible with a diagnosis of mild pleural effusion. There was no clinical evidence of overt bleeding manifestation. She had no features of focal neurology or meningeal irritation. The rest of the clinical examination was unremarkable, including optic fundi.

Initial evaluation revealed capillary blood glucose of high index (> 600mg/dL), hemoglobin of 10.3g/dl, white blood cells 4400 per mm^3^, with lymphocytes predominant and platelet count of 11000 per mm^3^. Transaminases were significantly elevated with aspartate aminotransferase (AST) of 7775U/l and alanine aminotransferase (ALT) of 1753U/l. Serum amylase and serum ferritin level were 200U/L and 72100ng/ml, respectively (Table 1). Venous blood gas analysis showed metabolic acidosis (pH 7.1, HCO- 14 mmol/L) with an anion gap and marginally elevated lactate. Both blood and urine ketone bodies were positive. The dengue NS1 antigen was positive, along with positive dengue IgM and IgG antibody. Blood and urine culture isolated no organism. Chest X-Ray revealed mild right-sided pleural effusion. Bedside ultrasound scanning demonstrated bilateral pleural effusion (right > left), thickened gall bladder wall with mild pericholecystic fluid, mild hepato-splenomegaly, and a minimum amount of free fluid in the abdomen with ultrasonically normal pancreas. Based on the clinical profile and results of an initial investigation, she was diagnosed with dengue hemorrhagic fever with diabetic ketoacidosis.

**Table 1:** Results of blood investigations; D1 (day of admission) and D11 (day of discharge) WBC-White blood cell, PCV-Packed cell volume, AST-Aspartate aminotransferase, ALT-Alanine aminotransferase, INR-International normalized ratio, APTT- Activated partial thromboplastin time.WBC-White blood cell, PCV-Packed cell volume, AST-Aspartate aminotransferase, ALT-Alanine aminotransferase, INR-International normalized ratio, APTT- Activated partial thromboplastin time.

Investigations	Normal reference range	D1	D2	D3	D4	D5	D6	D7	D11
WBC	4000-11000/mm^3^	4400	3600	3500	4000	4200	4800	4400	6800
Platelets	150000-450000/mm^3^	11000	13000	16000	30000	42000	48000	54000	210000
Hemoglobin	12-16 g/dl	10.3	10.4	10.6	10.6	10.5	11	11.2	11.7
PCV	35-45%	45.4	39	37.3	35	34.3	35	34.1	34.1
AST	8-20 U/l	7775	9348	7696	2186	1890	1300	678	85
ALT	13-35 U/l	1753	2151	1899	1276	970	680	432	46
Serum- ferritin	20-120 ng/L	72100		68000					
Serum amylase	80-180 U/L	200		158			151		
Blood urea	20-40 mg/dl	42	39		35		36	30	32
INR	0.8-1.3	1.8		1.9		1.6		1.4	1.2
APTT	19-30			32			28		
Serum creatinine	0.8-1.3		1.2				1.2		1.1

She was resuscitated with repeated boluses of normal saline to achieve the target hematocrit along with intravenous insulin infusion. She also was given one bolus of sodium bicarbonate. Her vitals were monitored very closely as per national guidelines on the management of dengue hemorrhagic fever published by the Ministry of Health, Sri Lanka [6]. Co-infection with other common viral (Epstein-Barr virus (EBV), cytomegalovirus, and influenza) and bacterial infections (typhus and leptospirosis) were excluded by performing appropriate investigations.

**Figure 1:**
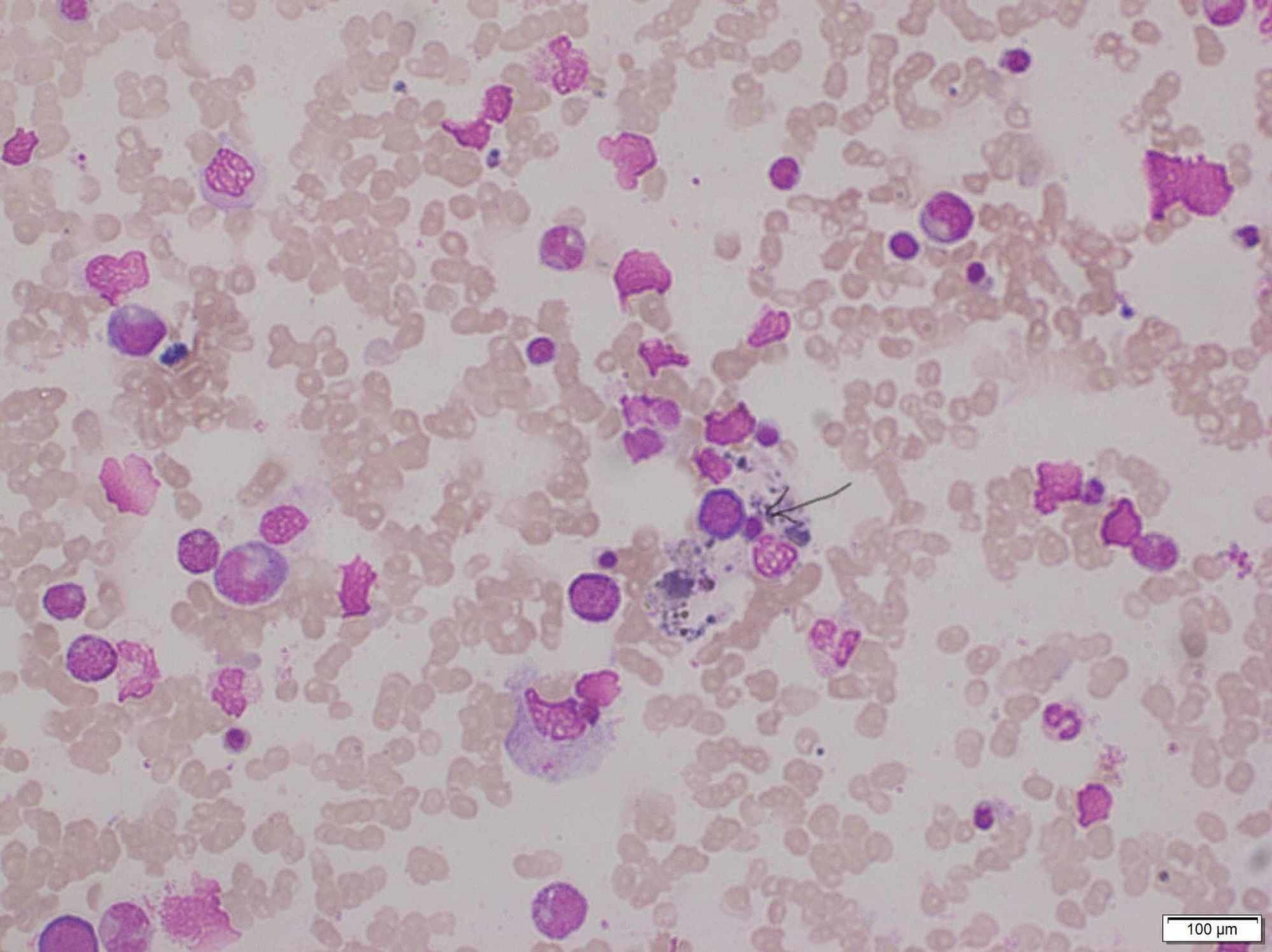
Bone marrow aspirate showing macrophage with marked hemophagocytic activity (arrowhead)

Although her vitals were returning towards normal, she showed no significant clinical improvement in terms of general wellbeing and appetite despite appropriate treatment. Her biochemical and hematology profile also showed a lack of adequate recovery. These findings, along with very high serum ferritin and splenomegaly, prompted an unusual but life-threatening complication of dengue hemorrhagic fever “hemophagocytic lymphohistiocytosis”. Subsequently, she underwent a bone marrow aspiration, which showed an increasing number of histiocytes with hemophagocytosis (Figure 1) compatible with a diagnosis of HLH. Then she was given a three-day course of intravenous methylprednisolone. After that, she started to improve dramatically, and finally, she made an uneventful recovery. She was sent home on day 11 following admission.

## Discussion

Dengue complicated hematological manifestations may present as disseminated intravascular coagulopathy (DIC), hemophagocytic lymphohistiocytosis (HLH), idiopathic thrombocytopenic purpura (ITP) and pancytopenia, with bone marrow biopsy revealing aplastic anemia, lymphadenopathy, hepatomegaly, and splenomegaly. HLH is possibly fatal in which uncontrolled proliferation and activity of macrophages in the reticuloendothelial system [7].

HLH encompasses a heterogeneous group of lethal, hyperinflammatory syndromes occurring in children and adults. It is patented by excess secretion of cytokines and unrestrained activation of macrophages and T cells, which causes persistent fever, hepatosplenomegaly, pancytopenia, hemophagocytosis, hyperferritinemia, and coagulopathy [8]. The diagnosis of HLH mainly depends on a cluster of clinical and biochemical abnormalities. If a patient meets five out of the following nine diagnostic criteria, that confirms the diagnosis of HLH. These include fever, splenomegaly, cytopenias (affecting two or more of three lineages in the peripheral blood), hypertriglyceridemia, hypofibrinogenemia, elevated ferritin, hemophagocytosis in bone marrow/spleen/lymph nodes, low or absent natural killer (NK)-cell activity, or elevated soluble CD25 (interleukin [IL]-2 receptor) [9,10]. As our patient had five out of these nine, the diagnosis of HLH was made.

The primary HLH is mainly due to underlying genetic irregularities; however, secondary HLH designates that the disorder is secondary to following conditions such as infection, autoimmune/rheumatologic, malignant, or metabolic conditions. Those who meet the criteria for HLH should be screened for triggering infection, EBV, cytomegalovirus, parvovirus B19, human immunodeficiency virus (HIV), and human herpes virus-6. Scrub typhus is common in Sri Lanka, which mimics dengue and triggers HLH [11]. The reason for splenomegaly is direct infiltration by lymphocytes and macrophages. The cytokine storm (high concentrations of tumor necrosis factor (TNF)-α and interferon (IFN)-γ) is the hallmark of HLH, which causes cytopenias and hemophagocytosis. A higher level of TNF-α decreases the lipoprotein lipase activity, which causes high triglyceride in the blood. Raised serum ferritin of more than 10,000μg/L has been established to be 90% sensitive and 96% specific for HLH [12,13]. Several cases of dengue complicated with pancreatitis have been reported [14]. In addition, dengue complicated with transient diabetic ketoacidosis has also been reported [15]. Our patient, with no past medical history, presented with DHF with diabetic ketoacidosis. Although the possibility of severe pancreatitis complicating diabetic ketoacidosis precipitated by the dengue virus was considered, our evaluation failed to demonstrate the evidence of acute pancreatitis. It was concluded that diffuse abdominal pain was probably due to diabetic ketoacidosis. However, the CT abdomen was not performed, and it is a limitation of this case report.

Early effective therapy reduces mortality in HLH. Treatment is intended to suppressing the hyperinflammatory state and immune dysregulation that leads to life-threatening organ damage. Treatment of hemophagocytic lymphohistiocytosis includes immune-suppressive and modulatory agents, bio­logical response modifiers, and stem-cell transplantation as well as treating the triggering condition [16]. Our patient was treated successfully with a short course of methylprednisolone, along with all other standard supportive therapies. She made an uneventful recovery.

## Conclusions

HLH is a rare but lethal complication of dengue hemorrhagic fever. Clinicians should suspect HLH as a possible complication in a patient with DHF having persistent fever, declining cell counts (pancytopenia), and elevated serum ferritin. Early diagnosis and prompt treatment would help to reduce mortality.
